# A Systematic Review of the Relationship Between Neonatal Hypoxic-Ischemic Encephalopathy and Long-Term Cognitive Outcomes: Where Do We Stand?

**DOI:** 10.7759/cureus.68227

**Published:** 2024-08-30

**Authors:** Mohammed Y Abusaleem, Muawia Elsayed Elkhider Ebrahim, Nazim F Hamed, Mohamed Farahat Mohamed Eladwy

**Affiliations:** 1 Pediatrics and Neonatology, Security Forces Hospital Dammam, Dammam, SAU; 2 Neonatology, Security Forces Hospital Dammam, Dammam, SAU; 3 General Pediatrics, Security Forces Hospital Dammam, Dammam, SAU; 4 Pediatrics, Security Forces Hospital Dammam, Dammam, SAU

**Keywords:** neonates, pediatrics, systematic review, neurodevelopmental, cognition, hypoxic-ischemic encephalopathy

## Abstract

This study aims to systematically review the existing literature on long-term cognitive outcomes in neonates with hypoxic-ischemic encephalopathy (HIE). A thorough search of PubMed, MEDLINE, and Embase was conducted to find studies that satisfied the inclusion requirements. Rayyan (Qatar Computing Research Institute, Doha, Qatar) was utilized during the whole operation. Our results included seven studies with a total of 521 patients and 247 (47.4%) were females. All of the included participants were assessed for the incidence of cognitive functions following hypothermia therapy. Newborns with significant HIE are at high risk for neurodevelopmental complications even in the absence of magnetic resonance imagining (MRI) abnormalities, such as poor performance score and hearing-language score, functional status, delayed language skills, emotional processing, sensory movement, learning, and memory. Independent of motor deficits, participants with a history of HIE are susceptible to issues with cognition and executive function during late childhood and adolescence. It is crucial to keep an eye on their intellectual development after infancy since cognitive dysfunction and memory problems might manifest subtly or not at all in the early years of life, but they can cause problems in later childhood and adolescence. Long-term follow-up research is also required to ascertain whether the enhanced cognitive outcomes will continue throughout adolescence. Even in cases where overt neuromotor abnormalities are not evident, children with watershed injuries on brain MRIs should be closely monitored to evaluate cognitive function, particularly language development.

## Introduction and background

Hypoxic-ischemic encephalopathy (HIE) is a type of brain injury that occurs when the brain is deprived of oxygen and blood flow, leading to damage and dysfunction of brain tissue. This condition can occur in newborns during childbirth, particularly if there are complications such as umbilical cord compression or placental abruption [1]. Neonatal encephalopathy (NE), on the other hand, is a broader term used to describe any type of brain dysfunction or injury that occurs in newborn babies. It can be caused by various factors such as infections, trauma, or metabolic disorders, in addition to hypoxic-ischemic events [2]. The main difference between the two is that HIE specifically refers to brain injury caused by oxygen deprivation and reduced blood flow, while neonatal encephalopathy encompasses a wider range of potential causes and manifestations of brain dysfunction in newborns [2].

One of the primary causes of newborn mortality and developmental psychomotor problems in children is HIE. In technologically sophisticated nations, the incidence of HIE is approximately 1-2 per 1,000 live births, while in less developed nations, it can reach up to 26 per 1,000 live births [1,3]. Even with the extensive use of therapeutic hypothermia, many infants will experience neurodevelopmental deficits, particularly when the HIE is severe [2]. In addition to various newborn morbidities, mortalities, and outcomes, survivors with HIE have demonstrated decreased cognitive functioning, behavioral adjustment, and school results during adolescence [3].

National (such as the American Academy of Pediatrics, British Association of Perinatal Medicine, and Saudi Neonatology Society) and global associations and professional societies (such as the International Society for Evidence-Based Neonatology (EBNEO)) have started a number of initiatives to promote and advance evidence-based neonatal healthcare [4]. In the field of neonatology, a growing number of clinical practice guidelines (CPGs) of varying quality are being published, despite the emphasis on the potential of CPGs to maximize patient outcomes and clinical practice [5,6].

While there is a growing body of research investigating the cognitive outcomes associated with neonatal HIE, there remains a lack of precise data regarding the percentage of NE cases specifically caused by prenatal hypoxia or ischemia. This gap in knowledge arises from the heterogeneous nature of newborn encephalopathy, which can result from factors that are not strictly hypoxic or ischemic, making it difficult to isolate cases of HIE for study purposes [7-9].

To effectively understand the neurocognitive consequences of neonatal encephalopathy, it is essential to conduct follow-up assessments during the school years. This is crucial because the development of cognitive skills continues throughout childhood, and certain cognitive functions may not be adequately assessed through neuropsychological tests at early ages. Consequently, normal developmental outcomes observed at 18 to 24 months following a neonatal hypoxic-ischemic insult do not necessarily preclude the emergence of subtle cognitive and behavioral challenges as the child matures [10-11]. Furthermore, mild cognitive deficiencies may only become apparent when children are faced with more demanding tasks during their educational journey [11]. This research aims to deepen the understanding of the relationship between neonatal HIE and the development of cognitive problems in children.

## Review

Methods

Preferred Reporting Items for Systematic Reviews and Meta-Analyses (PRISMA) standards were followed in this systematic review [12]. 

Search Strategy

A thorough search was carried out utilizing four important databases: PubMed, SCOPUS, Web of Science, and Google Scholar to locate pertinent content. We looked through databases with just English-language content, taking into consideration each one's particular requirements. We transformed the following keywords into PubMed Medical Subject Heading (MeSH) terms in order to locate the pertinent papers; "Hypoxic-ischemic encephalopathy," "Neonates," "Cognitive function," "Cognition," "Language," "Learning," "Neurodevelopmental," and "Behavioral functions." Three boolean firms, "OR," "AND," and "NOT," matched the specified keywords. Among the search outcomes were full-text English publications, free-to-download articles, and human trials.

To ensure the most relevant and up-to-date information, studies were chosen based on specific criteria. First, only studies investigating the connection between neonatal HIE and any type of cognitive impairment will be considered, regardless of the research design employed. Second, all studies must include participants with a confirmed diagnosis of HIE. To capture the latest advancements in the field, only recent studies published between 2000 and 2024 were included. Additionally, the studies must be available in English for analysis, and lastly, they must exclusively involve human participants.

Data Extraction

Rayyan (Qatar Computing Research Institute (QCRI), Doha, Qatar) [13] was used to check the search results in order to verify accuracy and detect duplications. To assess the relevance of titles and abstracts in the search results, inclusion and exclusion criteria were used. Papers meeting the inclusion criteria will be carefully scrutinized by reviewers. Any differences were worked out after careful consideration. A predefined data extraction template was employed to record pertinent study information, including titles, authors, study year, location, participants, gender, follow-up duration, administrated hypothermia therapy, and the main outcomes. We created a separate Excel sheet (Microsoft, Redmond, WA, USA) to assess the risk of bias of the included studies.

Data Synthesis Strategy

The information retrieved from pertinent studies was used to create summary tables that provide a qualitative assessment of the components and study outcomes. The best method for making use of the data from the included studies was identified after the data for the systematic review was gathered.

Risk of Bias Assessment

To assess the quality of the study, the Joanna Briggs Institute (JBI) [14] critical assessment criteria for studies reporting prevalence data were used. This tool consists of nine questions. Positive answers receive a score of 1, while negative, unclear, or irrelevant answers receive a score of 0. A total quality rating of less than four, five to seven, or more than eight was regarded as low, moderate, or exceptional quality, in that order. The quality of the study was evaluated independently by the researchers, and disagreements were settled by discussion.

To evaluate the risk of bias in the included randomized control trials, the Cochrane Collaboration Risk of Bias (ROB) tool [15] was utilized. The findings are displayed as a table with various color schemes. A significant risk of bias is indicated by red, a low risk by green, and an inability to assess the risk because of insufficient information is shown by yellow.

Results

Search Results

**Figure 1:**
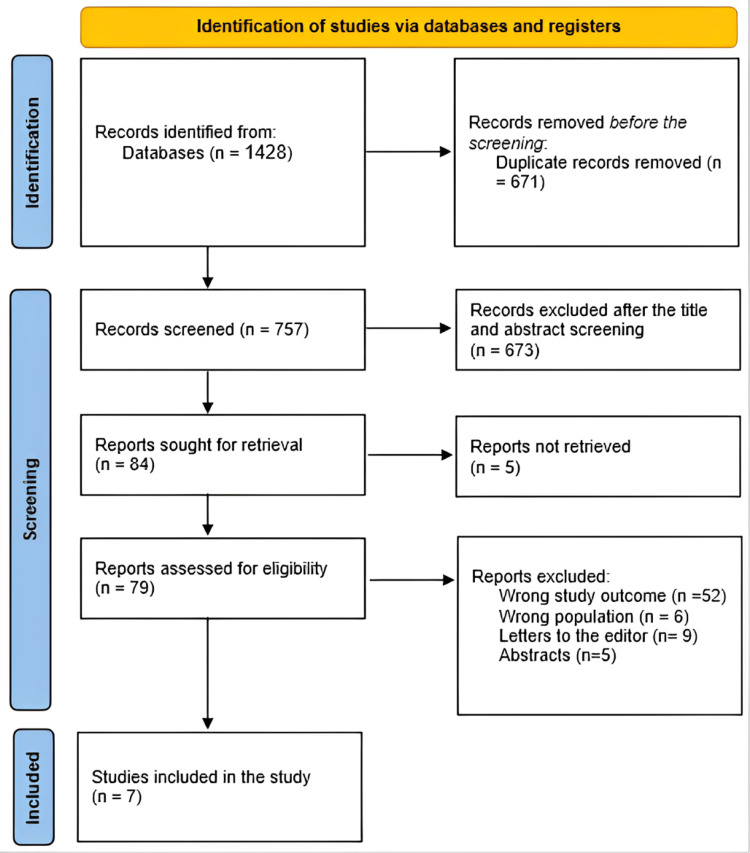
Study decision is summed up in a Preferred Reporting Items for Systematic Reviews and Meta-Analyses (PRISMA) diagram.

After 671 duplicates were removed, a total of 1428 study papers were found through the systematic search. After 757 studies had their titles and abstracts evaluated, 673 papers were discarded. Merely five things were not located out of the 84 reports that were required to be retrieved. Seventy-nine papers were screened for full-text assessment; 52 were rejected because the study results were wrong, six because the population type was inaccurate, nine articles were editor's letters, and five were abstracts. Seven research publications in this systematic review satisfied the requirements for eligibility. An overview of the procedure used to choose the research is shown in Figure 1. 

Characteristics of the Included Studies

The research publications' sociodemographic information is displayed in Table 1. Our results included seven studies [16-22] with a total of 521 patients and 247 (47.4%) were females. Three studies were prospective cohorts [18,19,22], two were randomized controlled trials (RCTs) [16,21], one was a case-control [17], and one was a cross-sectional study [20]. Two studies were conducted in the USA [16,19], two in the UK [17,21], one in Italy [18], one in Brazil [20], and one in China [22].

**Table 1:** The sociodemographic attributes of the participating populations. NM: not mentioned, RCT: randomized controlled trials

Study	Study design	Country	Participants	Mean age (Years)	Females (%)
Sutin et al., 2023 [16]	Prospective RCT	USA	58	32 ± 5.7	22 (38%)
Spencer et al., 2023 [17]	Case-control	UK	Patients (n = 31) and Controls (n = 32)	6 - 8	30 (47.6%)
Nadeem et al., 2011 [18]	Prospective cohort	Italy	35	NM	17 (48.6%)
Natarajan et al., 2014 [19]	Prospective cohort	USA	111	6 - 7	51 (46%)
Martinez et al., 2014 [20]	Cross-sectional	Brazil	70	NM	28 (40%)
Edmonds et al., 2020 [21]	RCT	UK	140	2	73 (52.1%)
Wang et al., 2023 [22]	Prospective cohort	China	44	NM	26 (59.9%)

Clinical Outcomes

The clinical features are displayed in Table 2. All of the included participants were assessed for the incidence of cognitive functions following hypothermia therapy. Newborns with significant HIE are at high risk for neurodevelopmental complications even in the absence of MRI abnormalities, such as poor performance score and hearing-language score [18], functional status [19], delayed language skills [20], emotional processing, sensory movement, learning, and memory [21,22].

**Table 2:** Clinical features and results of the included research. NM: not mentioned, NA: not applicable, CMRO2: cerebral metabolic rate of oxygen, HIE: hypoxic-ischemic encephalopathy, JBI: Joanna Briggs Institute

Study	Follow-up duration (months)	Hypothermia treatment	Main outcomes	JBI
Sutin et al., 2023 [16]	Follow-up started at the age of 18 months	Following hypothermia therapy	CMRO_2_ was positively linked with the motor and cognitive composite scores (P = 0.01 and P = 0.009, respectively; linear regression).	NA
Spencer et al., 2023 [17]	NM	Following hypothermia therapy	Whole-brain grey and white matter volumes are lower in children who experienced therapeutic hypothermia; hippocampus and thalamic volumes are correlated with functional outcomes (cognitive and motor).	Moderate
Nadeem et al., 2011 [18]	24	Following hypothermia therapy	Even in the absence of MRI abnormalities, newborns with significant HIE are at high risk for neurodevelopmental sequelae, such as poor performance score (P < 0.0001) and hearing-language score (P = 0.002).	Moderate
Natarajan et al., 2014 [19]	6 - 7 y	Following hypothermia therapy	A significant correlation was found between childhood impairment and poor independent functioning in children with HIE, as reported by their parents at 18 months of age.	Moderate
Martinez et al., 2014 [20]	24	Following hypothermia therapy	The language and age of the individuals indicate a statistically significant relationship that suggests children with neonatal HIE typically have delayed language skills, with the evidence increasing with age.	High
Edmonds et al., 2020 [21]	24	Following hypothermia therapy	Most patients without cerebral palsy (CP) have average Bayley-III scores, with mild cognitive delay in 5%, language delay in 4.2%, motor development in 1.3%, and motor development in 1.3%.	NA
Wang et al., 2023 [22]	24	Following hypothermia therapy	In terms of emotional processing, sensory movement, cognitive function, learning, and memory, neonates with moderate to severe HIE lag behind those with mild HIE.	Moderate

Risk Factors and Possible Mechanisms for This Relationship

Cerebral oxygen metabolism (CMRO2) was positively linked with cognitive composite scores [16]. One study found that whole-brain grey and white matter volumes are lower in children who experienced therapeutic hypothermia; hippocampus and thalamic volumes are correlated with functional outcomes (cognitive and motor) [17].

Risk of Bias

**Figure 2:**
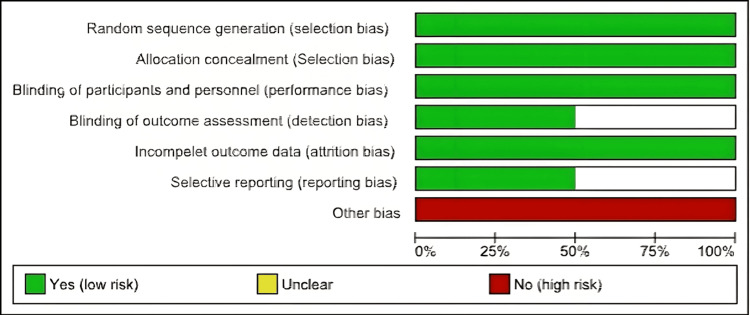
Risk of bias.

**Figure 3:**
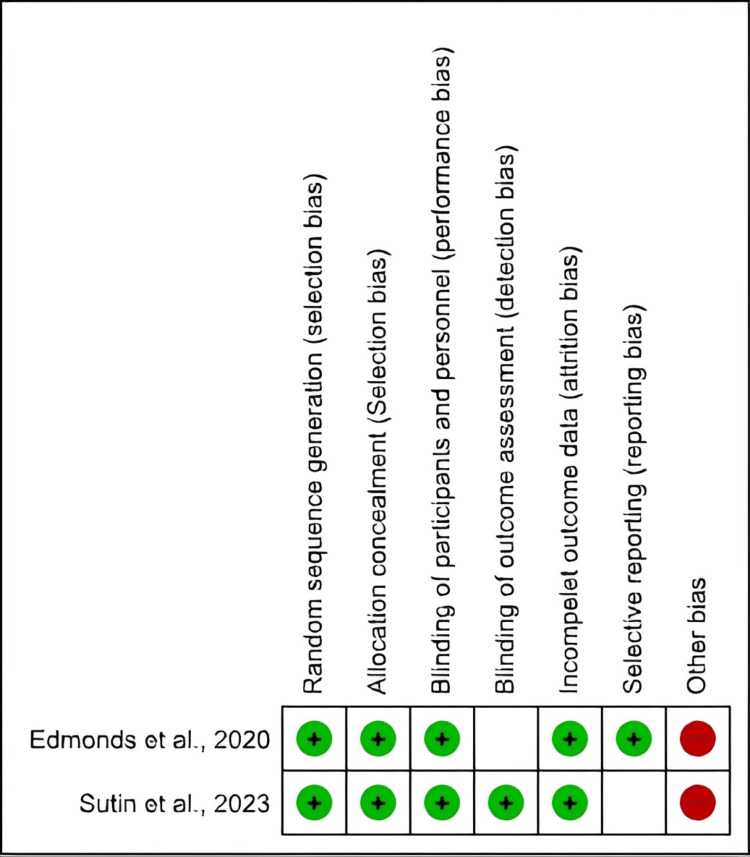
Risk of bias summary of the two included randomized controlled trials Sutin et al., 2023 [16] Edmonds et al., 2020 [21]

Five studies were non-randomized designs and assessed using the JBI tool; four were at moderate risk [17-19,22] and one was at high risk of bias [20] (Figure 2). We evaluated the RCTs using Cochrane and both were of high quality [16,21] (Figure 3).

Discussion

This study aims to comprehensively understand the link between neonatal HIE and the development of cognitive problems in children. All of the included participants in this review were assessed for the incidence of cognitive functions following hypothermia therapy. Therapeutic hypothermia for term neonates with moderate to severe HIE has been shown in several randomized clinical studies and a systemic review to result in a significant reduction in death or neurological damage by the time the babies are 18 to 24 months old [23,24]. The long-term benefits of hypothermia therapy on cognitive outcomes up until school age have, however, only been assessed in a small number of long-term follow-up clinical trials [25,26].

We found that newborns with significant HIE are at high risk for neurodevelopmental complications even in the absence of MRI abnormalities, such as poor performance score and hearing-language score [18], functional status [19], delayed language skills [20], emotional processing, sensory movement, learning, and memory [21,22]. A recent systematic review and meta-analysis conducted by Yinwen et al. found that the potential prevalence of serious neurodevelopmental impairments increased to 24% when patients with mild HIE were followed up until the age of three. The advantages of integrating therapeutic hypothermia as an auxiliary treatment with support treatment outweigh the drawbacks [27].

The degree of neonatal encephalopathy and the results of neurodevelopment are strongly correlated [28,29]. While infants who survive neonatal encephalopathy without motor deficits or with minor hypoxic-ischemic damage may have good outcomes in early childhood, children with severe neonatal encephalopathy often have a significant risk of delayed neurodevelopment. On the other hand, infants with a history of mild neonatal encephalopathy and survivors of prenatal hypoxic-ischemic injury without functional motor abnormalities frequently experience mild to moderate cognitive issues [30,31].

We found that CMRO2 was positively linked with cognitive composite scores [16]. Magnetic Resonance Spectroscopy (MRS) has previously looked into the connection between brain metabolism and neurodevelopmental performance in neonatal HIE treated with therapeutic hypothermia (TH). Measurements of metabolites such as lactate, N-acetylaspartate (NAA), or their ratio are commonly used by MRS to evaluate cerebral metabolism. MRS research has demonstrated that quantitative measurements of metabolites following TH are linked to catastrophic outcomes, which are defined as death or a two-year Bayley Scales of Infant and Toddler Development-Third Edition (BSID-III) composite score of less than 85 [29-31].

One study in this review reported that whole-brain grey and white matter volumes are lower in children who experienced therapeutic hypothermia; hippocampus and thalamic volumes are correlated with functional outcomes (cognitive and motor) [17]. IQ has been linked to hippocampal volume in both adults and children [32,33]. According to neuroimaging research, prenatal hypoxia causes hippocampal damage that lasts throughout development, impairing IQ and causing memory problems [34]. Furthermore, thalamic volume has been linked to verbal IQ in children who are usually developing [35], as well as visual memory function in patients with developmental amnesia resulting from hypoxic-ischaemic injury-induced hippocampal atrophy [36], aged 14 to 25 years.

The study had several limitations. The variability in study designs, methodologies, and diagnostic criteria for HIE among included studies can lead to inconsistent findings and limit the generalizability of results. Additionally, the heterogeneity in participant demographics, including differences in age, socioeconomic status, and associated comorbidities, may introduce confounding factors that impact cognitive outcomes, making it challenging to draw definitive conclusions. Furthermore, the reliance on retrospective data in certain studies can result in biases related to selective reporting and underrepresentation of milder cases of HIE. The follow-up duration also varies across studies, with many lacking long-term data that would provide a more comprehensive understanding of cognitive trajectories over time. Lastly, the potential for publication bias, where studies with significant findings are more likely to be published, may skew the overall conclusions of the review. 

## Conclusions

Independent of motor deficits, children with a history of HIE are susceptible to issues with cognition and executive function during late childhood and adolescence. Sustained observation may enable the early identification of children with cognitive issues, enabling timely interventions and providing the children with further educational evaluation and instructional support. Long-term follow-up research is also required to ascertain whether the enhanced cognitive outcomes will continue throughout adolescence. 
